# The impact of disrupted circadian rhythms and sedentary lifestyle on growth and health of children

**DOI:** 10.1097/MS9.0000000000005066

**Published:** 2026-04-24

**Authors:** Syed Shahmeer Ahmed, Nadia Mehmood, Shakeeba Zubair, Ahmed Asad Raza, Abedin Samadi

**Affiliations:** a Department of Medicine, Karachi Medical and Dental College, Karachi, Pakistan; b Department of Medicine, Aga Khan University Hospital, Karachi, Pakistan; c Department of Medicine, PNS Shifa Hospital, Karachi, Pakistan; d Department of Medicine, Jinnah Sindh Medical University, Karachi, Pakistan; e Department of Medicine, Kabul University of Medical Sciences Abu Ali Sina, Kabul, Afghanistan

**Keywords:** adolescent health, body mass index (BMI), childhood diseases, childhood obesity, children’s health, circadian rhythms, growth and development, health outcomes, health promotion, impact assessment, lifestyle factors, metabolic health, pediatric health, physical activity, physical inactivity, sedentary lifestyle, sleep disturbances, sleep hygiene, sleep patterns

## Abstract

Disrupted circadian rhythms and sedentary lifestyle patterns are increasingly recognized as significant contributors to impaired growth and adverse health outcomes in children and adolescents. Rising screen exposure, reduced physical activity, and irregular sleep schedules are associated with declining health-related fitness and increased risk of obesity and noncommunicable diseases. Circadian rhythms regulate sleep–wake cycles and hormonal pathways essential for metabolic balance and normal development. Excessive use of smartphones, gaming, and digital media – particularly at night – leads to sleep dysregulation and altered melatonin and growth hormone secretion, potentially affecting physical and neurocognitive growth. Sedentary screen-based behaviors show strong negative associations with physical fitness, body mass index, and psychosocial well-being. Regular physical activity acts as an important circadian synchronizer, improves sleep quality, and supports metabolic and musculoskeletal health. Although technology contributes to inactivity, app-based and gamified interventions may help promote movement, although long-term sustainability remains uncertain. Preventive strategies should emphasize sleep hygiene, structured daily routines, parental counseling, controlled screen time, and school-based physical activity promotion. Addressing both circadian disruption and sedentary behavior is essential for optimizing pediatric growth, metabolic stability, and long-term health outcomes.

## Global burden of physical inactivity and declining adolescent fitness

The fourth-largest behavioral risk factor for disease and death is physical inactivity. In many nations, the proportion of individuals who are physically inactive is increasing, which has an impact on the incidence of noncommunicable illnesses and the general health of the world’s population^[^1^]^. In recent years, adolescent Health-Related Fitness has decreased in accordance with physical education classes. All young people had declines, but those who had normal body mass index (BMI) and low fitness levels were significantly affected.

## Screen-based sedentary behavior and its impact on health

Policymakers could improve PE in schools to encourage activity and stop the fitness and health of future generations from declining in order to reach the majority of young people^[^2^]^. The findings of a trial demonstrated a detrimental association between playing video games and men’s health, social interactions, and physical exercise. Electronic games and physical activity had the strongest negative connection, followed by social ties and BMI. Health had the worst performance^[^3^]^. Beyond its impact on physical inactivity, prolonged screen exposure may also interfere with biological circadian rhythms.

## Circadian rhythm disruption and sleep dysregulation in adolescents

In addition to promoting sedentary behavior, digital screen exposure particularly during nighttime hours can disrupt circadian rhythms and sleep regulation. The circadian clock regulates the sleep-wake cycles, which are crucial for maintaining both physical and mental health, as well as whole-body homeostasis^[^4^]^. Day/night cycles and seasonal rhythms are activities that have been well preserved through evolution^[^5^]^. The use of smartphones for entertainment purposes, e.g., electronic games, social websites, and apps like Netflix and amazon prime etc., has impacted the sleep cycle of adolescents. These disturbances may further influence hormonal pathways involved in growth and metabolic regulation.

## Growth hormone, melatonin, and the role of exercise in circadian regulation

The principal physiological factors that control Growth Hormone (GH) secretion include the brain endogenous rhythm, sleep, stress, physical activity, and signals from the body’s metabolism and nutrition^[^6^]^. There is evidence that the pineal hormone melatonin controls the amount of GH that is secreted^[^7^]^. In all lifespans, but especially in aging, exercise is a crucial nonphotic Zeitgeber that synchronizes the circadian clock, regulates sleep depth, and modulates muscle physiology. Notably, there is ongoing debate on the direct impact of exercise on the production of endogenous melatonin, perhaps as a result of inconsistent melatonin measurements in the blood or saliva. Salivary melatonin levels in males were measured in the late evening, and they were shown to be negatively related to physical activity time because they were greater in the morning session compared to the late afternoon session of steady-state jogging. This suggests that morning fitness may be associated with normal sleep patterns^[^8^]^.

## Technology as both the problem and the potential solution

Therefore, it is essential to look for solutions to decrease the screen time to improve the sleep cycle of adolescents and work on ways to increase their physical activity. Apps for smartphones are now acknowledged as a feasible and promising strategy to improve adherence to physical activity recommendations. Globally, there are more active mobile phones than people^[^1^]^. A smartphone game called Pokémon Go employs augmented reality, and players are encouraged to go outside and travel long distances in order to capture the Pokémon. Initial studies reported short-term increases in daily step counts among users; however, follow-up research suggests that these behavioral effects decline over time, highlighting the challenge of sustaining long-term physical activity through digital interventions. While integrating physical exercise with current technology appears promising, it is obvious that inventive approaches must be investigated if a longer-term impact is to be realized^[^9^]^.

## Parental involvement and behavioral interventions

Parental involvement is crucial in shaping children’s sleep habits, screen exposure, and physical activity behaviors. Studies indicate that parental regulation of bedtime routines and screen time is associated with improved sleep quality and healthier behavioral outcomes in children and adolescents. Establishing consistent sleep schedules and limiting evening digital device use may help maintain circadian rhythm stability and support normal growth and metabolic health.

Educational interventions and counseling for parents can raise awareness about the health risks of irregular sleep patterns and excessive sedentary screen use. Encouraging participation in sports, outdoor play, and structured physical activities may further help reduce sedentary behavior and promote overall well-being in youth^[^10,11^]^.

## Conclusion

The convergence of sedentary digital lifestyles and disrupted circadian rhythms represents an emerging threat to pediatric health worldwide. While technology contributes to behavioral inactivity, it may also provide innovative tools to promote healthier habits when used responsibly. Addressing these lifestyle patterns through parental education, school-based interventions, and public health strategies is essential for protecting children’s growth, metabolic health, and long-term wellbeing.

To ensure methodological transparency and ethical compliance in manuscript preparation, we adhered to the recently developed TITAN (Transparency in the Reporting of Artificial Intelligence) 2025 guidelines, which outline standards for responsible AI use in biomedical writing and publishing^[^12^]^.

## Data Availability

No datasets were generated or analyzed during the current study.
